# Diagnostic and management challenges of late chest wall mass following implant-based breast reconstruction: a case report

**DOI:** 10.1080/23320885.2025.2610546

**Published:** 2025-12-31

**Authors:** Kathryn Howard, Garrett M. Minor, Alisha B. Paranzino

**Affiliations:** a College of Medicine, University of Kentucky, Lexington, KY, USA; b Division of Plastic Surgery, University of Kentucky, Lexington, KY, USA

**Keywords:** Chest wall mass, late hematoma, implant-based breast reconstruction, case report

## Abstract

Peri-prosthetic late hematoma following breast implant procedure is defined as a hematoma presenting longer than 6 months after operation and is a rare complication with sporadic cases reported throughout literature. We present a case of an 85-year-old patient who developed a spontaneous late hematoma nearly 20 years following implant-based breast reconstruction and adjuvant chemotherapy to illustrate the importance of maintaining a wide differential when approaching a chest wall mass that cannot be biopsied in a patient who has previously received breast implants. MRI revealed a peri-implant effusion with a heterogeneous mixed signal partially enhancing mass measuring 3.2 x 4.3 x 1.7 cm, posterior to the left breast implant. The patient had bilateral Mentor smooth, round, silicone, 350 cc implants in the submuscular plane. Ultrasound-guided biopsy was attempted and unsuccessful due to inability to displace the implant and access the mass, indicating the need for an open biopsy. The patient underwent radical left chest wall mass excision of the posterior implant capsule, removal of the left implant, and closure of the anterior capsule. Final pathology confirmed the diagnosis of organized hematoma. Immunophenotyping flow cytometry was utilized to rule out BIA-ALCL or BIA-SCC. Our case is unique in that biopsy was unable to be obtained given retro-implant position of the mass and that the diagnosis and etiology of late hematoma formation following smooth round silicone implants has been infrequently discussed in literature. Providing a comprehensive workup considering patient history, physical exam findings, imaging, and pathology ensures a wide differential optimizing patient outcomes.

## Introduction

Implants are commonly used in breast surgery for both cosmetic and reconstructive purposes [1]. Patients can experience adverse effects associated with breast implants, which are separated into early and late complications based on the time of operation [1]. Early complications can include infection, hematoma, seroma, mastectomy flap skin necrosis, wound dehiscence, and implant extrusion and occur within weeks following the procedure [2]. Late complications occur within months to years and include capsular contracture, implant leakage or rupture, breast implant associated anaplastic large cell lymphoma (BIA-ALCL) and breast implant associated squamous cell carcinoma (BIA-SCC) [2,3].

Hematoma is a well-known complication most commonly occurring during the early postoperative period. Peri-prosthetic hematomas present in 2–10.3% of patients following breast implants and typically occur within 3 days following the operation [3]. Possible etiologies have been identified as inadequate hemostasis, trauma, or coagulopathy [4]. A peri-prosthetic late hematoma following a breast implant procedure is defined as a hematoma presenting longer than 6 months after operation and is a rare complication with sporadic cases reported in literature [2,3]. The etiology of late hematoma formation is not clear, but previous reports have identified several possible causes including direct trauma, clotting disorders, anticoagulant use, physical strain, post-chemotherapy immunodeficiency, use of corticosteroids at the time of implantation, or the friction force between the textured prosthesis surface and highly vascular surrounding tissue [2,3]. A previous study conducted in 2021 included a literature review and identified only 30 case reports of late hematoma following breast implants with 18 for aesthetic purposes and 12 for breast reconstruction [3].

The clinical presentation of a late progressively enlarging breast following a breast implant procedure is extremely rare [5]. The typical symptoms of late hematoma are breast pain and swelling with physical exam revealing breast asymmetry and tenderness on the affected side [6]. Less common symptoms include skin discoloration and discharge from a sinus tract [6]. Typically, systemic signs of infection such as fever, fatigue, and malaise are absent [6].

We present a case of an 85-year-old patient who developed a spontaneous late hematoma nearly 20 years following implant-based breast reconstruction and adjuvant chemotherapy to illustrate the importance of a wide differential including sarcoma, BIA-ALCL, BIA-SCC, recurrent breast cancer, benign late seroma, and hematoma when approaching a chest wall mass that cannot be biopsied in a patient who has previously received breast implants.

## Case report

An 85-year-old Caucasian female presented to our institution for evaluation of a left breast mass. The patient reported that over the last several months she noticed progressively worsening firmness, pain, and deformation of her left breast with a pulling sensation at her left axilla.

The patient has a history of right breast cancer diagnosed in 2005, categorized as stage IIB hormone receptor positive invasive ductal carcinoma. At the time of her diagnosis, she underwent bilateral skin-sparing mastectomy with positive right sentinel lymph node biopsy followed by immediate reconstruction with subpectoral tissue expander placement. She subsequently underwent implant exchange with Mentor smooth round silicone 350 ml high profile implants. The patient completed adjuvant chemotherapy (Adriamycin/Taxol), five years of aromatase inhibitors, and did not require radiation. She has not undergone replacement of her implants in the past 20 years. She is a nonsmoker, nondiabetic, and does not take anticoagulation. She drinks approximately one glass of wine per night. She has a past medical history of hypertension, mitral valve prolapse, osteoarthritis, and skin cancer. She has a history of numerous corticosteroid injections to the right knee without improvement of her symptomatology.

**Figure 1.**
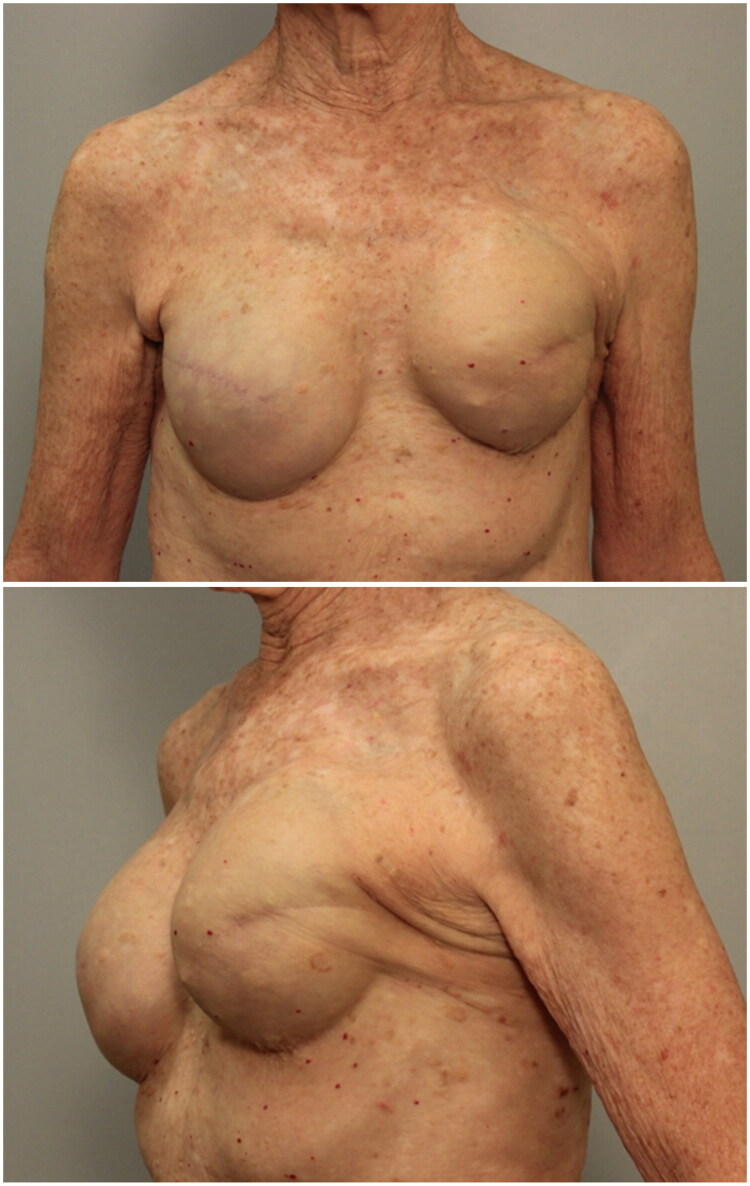
Preoperative photos demonstrating elevation of the left breast implant.

At the time of her presentation, clinical exam revealed evidence of breast asymmetry. The left breast was noted to be superiorly displaced on the chest wall with firmness and tenderness; there was no appreciable difference in volume (Figure 1). The left inframammary fold was noted to be 2 cm higher than the right. The patient denied any fever, chills, erythema, discharge, unintended weight loss, change in appetite, night sweats, or new lumps/masses. She also denied any recent or remote history of breast trauma.

An MRI was obtained to evaluate the integrity of the implant. The patient was found to have an intact implant and a small peri-prosthetic effusion with a heterogeneous mixed signal. Notably, there was found to be a partially enhancing mass measuring 3.2 × 4.3 × 1.7 cm, posterior to the left breast implant. Breast Implant Reporting and Data System (Bi-RADS) criteria was utilized to place the mass in category 4, indicating a suspicious abnormality. MRI did not reveal right or left axillary or internal mammary lymphadenopathy. Ultrasound-guided biopsy was attempted and was unsuccessful due to inability to displace the implant and access the mass, indicating the need for an open biopsy.

Given the inability to access the mass *via* imaging guided biopsy, as well as the suspicious features, the patient was subsequently referred for multidisciplinary tumor board discussion. Following referral, recommendations were made for implant removal and open biopsy in conjunction with our surgical oncology colleagues.

**Figure 2.**
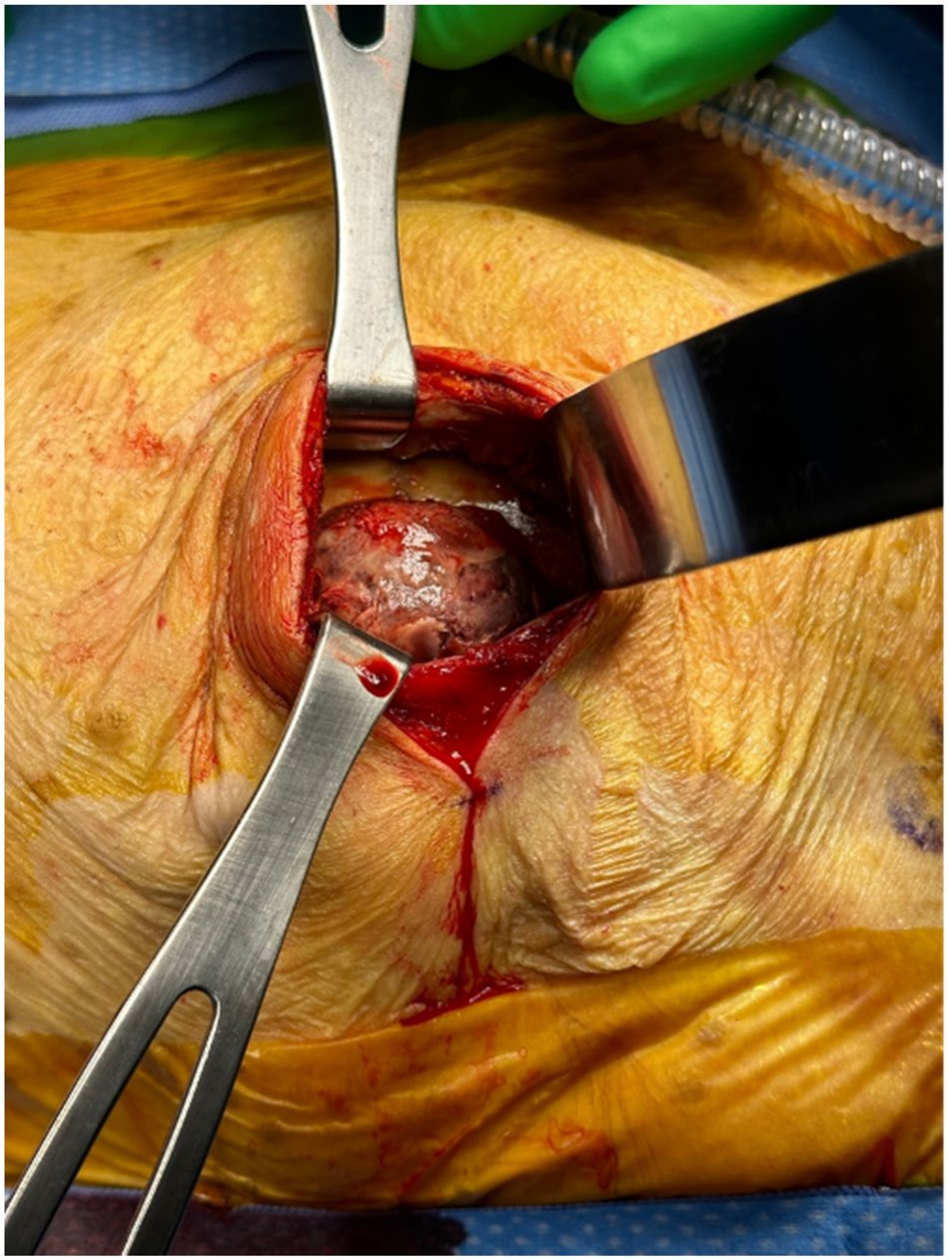
Friable mass adherent to posterior capsule and chest wall.

The patient was taken to the operating room, and the existing mastectomy incision was used for access to the implant pocket. The pectoralis muscle was split along the orientation of the muscle fibers, and the anterior implant capsule was encountered. When the capsule was incised, we immediately encountered 20 mL of sanguineous periprosthetic fluid which was removed and sent for cytology. The implant was removed and confirmed to be intact and grossly unremarkable. The aforementioned mass, measuring approximately 3 × 4 cm, was found to be fixed to the chest wall, invading the posterior capsule, and was noted to be quite friable (Figure 2). The surgical oncology team performed a radical resection involving 1 cm margins to a depth of underlying periosteum and intercostal muscle fascia.

Total capsulectomy was discussed with the multidisciplinary tumor board. However, given that chest wall sarcoma was on the differential, the surgical oncology team preferred that the mass be accessed *via* anterior capsulotomy and removal of the implant to limit potential disturbance to the mass that could occur with total capsulectomy. Following radical resection, we discussed performing further capsulectomy, however the surgical oncology team preferred that the anterior capsule be closed to limit contamination of cancer cells within the surrounding tissues in the event that final pathology demonstrated sarcoma.

After resection, meticulous hemostasis was ensured utilizing electrocautery under normotensive conditions. The remainder of the capsule was noted to be grossly normal. A 15 French Blake drain was placed within the remaining implant pocket and brought out in line with the incision. The anterior capsule was imbricated with 2-0 PDS to isolate the tumor cavity while we await pathology. The soft tissues were closed with deep dermal 3-0 Monocryl followed by a running subcuticular 4-0 Monocryl. The drain was secured with 2-0 Silk suture.

Final pathology confirmed the diagnosis of organized hematoma and focal fat necrosis with no tumor seen. The pathology report described a 5.2 × 4.2 × 1.8 cm ellipse of oriented, necrotic tan skin featuring a central, bulbous, 3.5 × 3.0 × 1.6 cm tan lesion extending to within 0.2 cm of the superior, 0.9 cm of the lateral, 0.3 cm of the inferior, and 1.0 cm of the medial margins. Cut section revealed a 3.7 × 1.6 × 3.0 cm homogenous, tan-red hemorrhagic mass extending to within 0.1 cm of the deep margin. Peri-implant fluid was collected, and immunophenotyping flow cytometry was utilized to rule out BIA-ALCL or BIA-SCC. Flow cytometry interpretation was negative and showed a mixed lymphocyte population with no immunophenotypic evidence of clonal or aberrant populations.

**Figure 3.**
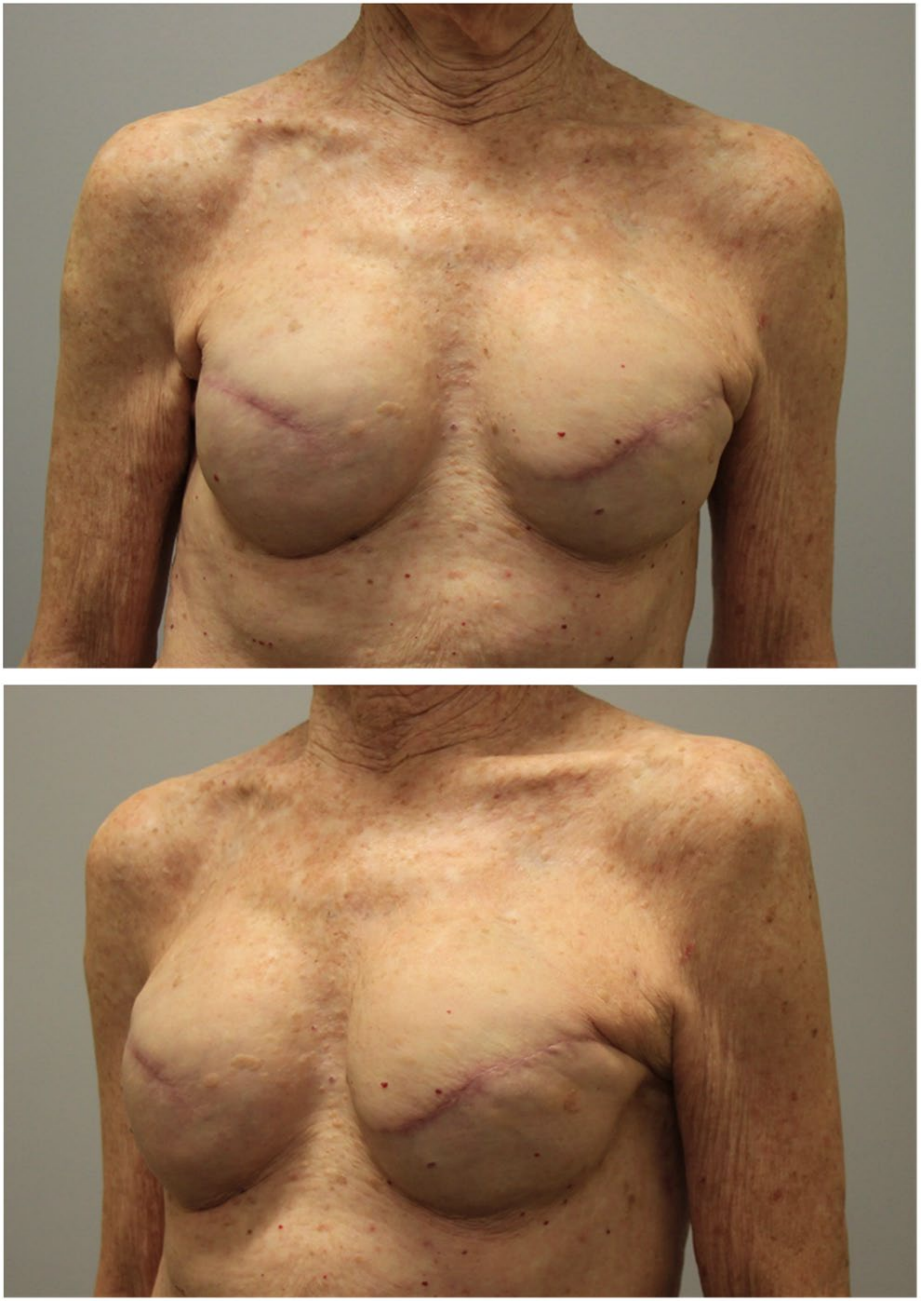
Postoperative photos demonstrating restored symmetry after bilateral implant replacement.

Postoperatively, the patient developed a seroma after drain removal which was managed conservatively. She elected to proceed with placing a new implant in the left breast. Given that her right implant was nearly 20 years old, it was also exchanged for symmetry. Approximately 6 weeks after her initial biopsy, she underwent bilateral implant placement with Sientra smooth round silicone 350 mL high profile implants. The patient required capsulotomy and capsulorrhaphy of the left breast for symmetry. During this procedure the biopsy site was noted to be well healing with a small region of exposed perichondrium. A drain was placed on the left side, and the patient did not experience recurrent seroma. At 6 months postoperatively, the patient is well healed with no complications. Postoperative photos demonstrate restored symmetry (Figure 3).

## Discussion

Peri-prosthetic late hematoma following breast implant placement is a rare occurrence documented sporadically throughout literature, primarily through case reports. Documentation of these cases is important to increase awareness, as well as to contribute to the determination of the etiology. In this case, there was not a suspected inciting event for late hematoma formation. One of the most critical points is the clinical approach to ensure optimal patient safety. Appropriate workup was completed prior to taking the patient to the operating room for open biopsy due to the wide differential and inability to obtain tissue diagnosis prior to surgery. The differential included BIA-ALCL, BIA-SCC, breast cancer recurrence, and sarcoma.

The differential diagnosis of delayed breast asymmetry after implant placement must be broad and include breast implant associated cancers. These can present clinically with a peri-implant effusion, unilateral swelling, pain, or capsular contracture [3]. The estimated risk of BIA-ALCL in patients with textured breast implants in the United States is 1:913 [7]. The diagnosis is confirmed through immune-histological and cytological analysis of peri-prosthetic fluid [8]. Spontaneous breast seroma presentation following breast implants that occurs more than 1 year after surgery should be considered suspicious for BIA-ALCL, as it is estimated to occur in 9–13% of delayed seroma presentations [3]. Late seroma is rare with an incidence of 0.88–1.84% and occurs at least 12 months after surgery [3]. In this case, the presentation was less consistent with classic unilateral breast swelling and more consistent with a discrete chest wall mass which could be consistent with BIA-SCC.

BIA-SCC is a rare malignancy with a lifetime risk of 1:164,884 [9]. Due to the growing data pool, treatment guidelines have yet to be established [10]. Diagnosis of BIA-SCC has occurred 15–42 years following breast implant placement [10]. Cases have occurred in patients with silicone, saline, textured, and smooth implants [11]. Patients have presented with symptoms including swelling, pain, lumps, and skin changes [10]. When evaluating a patient presenting with changes in their breast, operation should not occur without appropriate preoperative assessment to determine the likelihood of breast implant associated malignancy [10].

The detection of breast cancer recurrence is done through physical examination and directed imaging studies with physical exam being the most common method for initial detection of locoregional recurrence [12]. Detection of locoregional breast cancer recurrence is not affected by the presence of a breast implant [12]. Additionally, previous studies have not shown a difference in incidence of locoregional recurrence in patients who had breast reconstruction compared to those who did not [12]. Treatment of breast cancer recurrence in patients with breast implants includes surgical excision, chemotherapy, radiotherapy, and hormonal therapy [12].

Sarcoma is a broad group of cancers that originate from connective tissue including bone and muscle. Fine needle aspirate may be sufficient for initial diagnosis; however, core needle biopsy is typically required for more extensive histologic analysis. If a core biopsy is unsuccessful for pre-operative diagnosis, an excisional biopsy can be used [13]. There is a longstanding concern that tissue sampling of sarcoma may result in ‘seeding’ of cancer cells along the biopsy tract. Thus, any tracts (including drain placement) must be well defined, and attempts are made to confine interventions by anatomic compartments which would be more amenable to re-excision.

Breast sarcoma originates from the mesenchymal tissue of the breast and accounts for <1% of primary breast malignancies [14]. Most commonly, patients present in the fifth or sixth decade of life with a unilateral well defined painless mobile breast lump that is rapidly growing [13]. MRI is a useful tool for detecting breast sarcoma with high sensitivity, but slightly lower specificity [13]. Management of breast sarcoma includes wide local excision or mastectomy [13]. In this case, there was a higher suspicion for chest wall sarcoma than breast sarcoma due to the patient’s history of mastectomy. Soft tissue sarcomas of the pectoralis major muscle can closely resemble breast lesions on clinical examination [15]. Optimal treatment of sarcoma is characterized by involvement of a multidisciplinary team including experienced sarcoma surgeons, pathologists, radiotherapists, and medical oncologists prior to the beginning of treatment [13].

A previous systematic literature review conducted in 2021, identified 30 case reports of late hematoma following breast implants with 18 for esthetic purposes and 12 for breast reconstruction [3]. The onset of late hematoma varied from 4 months to 41 years after surgery [3]. The most commonly described etiology was mechanical friction between the textured surface of the implant and the highly vascular capsule resulting in capsule microfractures [3]. Compared to prior cases reported in the literature, our case is unique in that our patient has a high chronologic age and age of implant following placement for breast reconstruction. Additionally, our case provides an example of late hematoma presentation following the use of smooth round silicone breast implants for breast reconstruction which has previously been discussed less frequently than late hematoma presentation following the use of textured implants.

A previous study documented the occurrence of late hematoma in five patients who received smooth, round silicone implants [16]. In each case, the patient presented with a progressively enlarging breast [16]. The suspected etiology appeared to be from multiple and recurrent episodes of bleeding [16]. This presentation varies from our case because our patient did not present with an appreciable difference in volume between her breasts, instead the affected breast was noted to be superiorly displaced on the chest wall. A plausible mechanism for late hematoma formation in our case is the fragility of the capsular vessels due to the advanced patient age and her history of hypertension increasing the chance of microtrauma. This case was diagnostically challenging because imaging studies revealed a suspicious mass, but image guided biopsy was not possible because of the retro-implant position of the mass. A definitive diagnosis was unable to be made preoperatively, which resulted in a wide intraoperative differential including BIA-ALCL, BIA-SCC, recurrent breast cancer, and sarcoma. A broad differential with comprehensive workup is key when approaching an atypical, delayed presentation of a post-implant complication as rare peri-implant malignancies can mimic benign complications.

Ultrasonography and MRI are useful diagnostic tools but are unable to definitively distinguish late hematomas from implant ruptures [3]. In this case, the diagnosis of late hematoma was ultimately confirmed through surgical exploration and pathology. This emphasizes the need to maintain a high level of clinical suspicion for abnormal complications, like late hematoma, particularly in cases with unusual presentation or delayed onset.

## Conclusion

We present a unique case of a unilateral chest wall mass in the setting of previous breast cancer, confirmed after surgical exploration and pathology to be an organized hematoma occurring nearly 20 years following implant-based reconstruction. Our case is unique in that biopsy was unable to be obtained given retro-implant position of the mass indicating the need for a broad differential. It is essential to approach each clinical case as a unique situation and provide a comprehensive pathological workup considering patient history, physical exam findings, imaging, and pathology to ensure a variety of possibilities are considered to optimize patient outcomes particularly in the case of an atypical or late presentation. A thorough workup may expedite the recognition of clinically significant rare complications.
